# Our Dental Literature

**Published:** 1881-03

**Authors:** L. Ashley Faught


### ARTICLE V.

### *Our Dental Literature.*

The following remarks of Prof. L. Ashley Faught, D. D. S., before the Odontological Society of Pennsylvania, are interesting and to the point:

Convinced that our profession might be benefited by a retrospect of its literature, I presume to offer some remarks upon this subject from the critic standpoint. I am well aware that such position is unenviable, but a work wrought for my own benefit has developed so many facts of peculiar interest that I feel impelled to make a statement of them to the profession.

For several years I have followed and compared the writers and writings of the day to note the progress of dentistry. In my studies of the writings it seemed to me that chaff exceeded wheat; while with writers it seemed that echoes exceeded the voices.

Upon the cover of one of our journals we find these words: "Observe, compare, reflect, record." A glorious and effective motto. How has it been heeded by the profession? The word "record" seems to have been all that has been noticed. The suggestive gradation from observation to comparison, from comparison to reflection, has been almost totally ignored. Record! Record!! To record everything, valuable or valueless, and publish it too, has so become the act of our contributors that doctrine and originality of idea consequent upon investigation is fast becoming obsolete. If they would but ponder on the quadruple motto as an entity, and grasp its powerful truth, I am sure that the savor of freshness would greet us as we perused the pages of future dental journals; for observation would open the way to hidden and unknown things; comparison would prevent the repeated publication of ideas already well expressed; and reflection would so imbue the dental mind with the sanctity of the space in the media of publication, and with the value of the time of our societies, that no one would dare to trifle with them as at present, but would only occupy as he had something which was new and worthy of presentation.

I admit that repetition of old ideas was once needed, but claim that the day has long passed. In earlier times our journals were the sole disseminators of knowledge, but now we have adequate colleges to train those entering upon active practice. These should be, as it were, the primary and secondary schools, so that the journals might become the normal and university. The students of the profession should be trained by our present college chairs, or by the establishment of a new one—that of dental literature—and by adequately prepared text-books, in all that has been presented to the profession, by all writing up to the date of their graduation. It should be the work of the professors or professor to thus send them forth fully acquainted with the past, and fully prepared to comprehend and contribute to the writings of the future. Journals then need only contain that which is new since their last and up to their immediate date of issue. Under this condition of affairs practitioners would eagerly look for them, and, turning their pages over and over in study, would thus keep upon the crest of the wave and ever be ready for progressive work. The occupants of professorial positions, securing this material as it in turn became old, would incorporate it in their lectures to the coming recruits. Thus would the wheels of dentistry assuredly roll onward.

In order that this retrospect may not become voluminous, I will confine my observations to the *Dental Cosmos*, as a typal journal and of largest circulation, and to the years from 1872 to 1880 inclusive.

Carefully enumerating and tabulating, I have discovered that of the twelve thousand dentists in the United States only three hundred and thirty-four have, in the nine years, contributed anything to its pages, and even this small number is attainable only by including every member reported as having made remarks in a dental society! Of this contributing, twenty-five have done more than one-third of the work, and fifteen of these occupy positions as teachers in dental institutions. From such we naturally expect to hear, as they are supposed to be gathering material in their experimental work as instructors. Can it be possible that there are only ten active minds in the private ranks of the profession of dentistry? And yet it seems but reasonable to infer that *workers* will desire the widest promulgation of their labors.

Making an exception of two serials, which were copyrighted for after publication as text-books, I have also found that the number of subjects thus presented during the above stated time is one hundred and thirteen. They may be classified as follows: Speculative, sixty-eight; relating to materials and drugs twenty-five; manipulative, fourteen; pathological and therapeutic, six. By this classification we see that the bulk of matter,—speculative,—even though it were handled in a masterly manner, is at best of questionable value.

The subjects receiving the most attention were: "Sixth-year Molars," "Eclectic Dentistry," "Neuralgia," "Caries," "Conservatism," "Doctor," "Pulpless Teeth," "Exposed Pulps," "Sensitive Dentine," "Contour Filling," "Creosote" and "Amalgams." What a record is this when we consider the broad field of Dentistry!

Carefully analyzing the papers and comparing them the following facts have been elicted:

*First.* It is noted that the same practitioners, when writing at intervals on the same subject, not only state the same ideas, but in many instances present them by phraseology absolutely identical.

*Second.* In views relating to special subjects advanced by different practitioners a marked similarity is constantly observable. For instance, in articles upon "Exposure of the Pulp," the same ideas are stated and restated by different persons. Almost all papers upon it consist of a stereotyped discussion of merits of creosote, oxychloride of zinc, and Hill's stopping, with an occasional reference to the temperament of the patient, the condition of the pulp at time of capping, and the length of time of probation.

*Third.* It is seen that a large bulk of the matter in papers published is taken from text-books.

*Fourth.* It can be shown that many of the views advanced as dogmatic truth are so totally unscientific as not only to subject the enunciator but also his profession to open ridicule. For instance, in September, 1878, a gentleman is reported as having said, ". . . our modern amalgams have not the preservative quality of the old, which was due to the copper salts which they contained;" while a request in the October number, that he would state the names of these salts, and explain why they could not be incorporated in the less preservative modern amalgams, failed to elict a reply. Again in November, 1880, a gentleman is reported as having said, ". . . and in all these cases carbolic acid ought to be placed in the cavity to disinfect the carious dentine and coagulate the protoplasm weeping from the fibres ot living matter in the exposed dentinal canaliculi;" while it is believed that the scientific world of to-day does not recognize the use of the word protoplasm, which in former years was employed to express the contents of a vegetable cell, the granular matter around the so-called nuclei of muscles, and the moving matter of the colorless blood corpuscles. For the sake of showing further error let us apply these ancient meanings to the words used. The gentleman certainly did not refer by it to the first two. He must therefore have meant the latter one. This is incapable of coagulation, and, as living matter itself, possesses not the possibility of weeping from other living matter. Other points in the expression quoted, besides these mentioned, render the sentence extremely vague, mythical, and questionable.

In conclusion, I trust that those who have been reading with the blissful feeling that they were progressing will "reflect" upon the points I have mentioned, and, remembering that if our journals do not bring us new facts in the future the fault is not with the editors but with ourselves, will stop this heterogeneous writing on anything and nothing, and as a first principle resolve to publish no paper or remark unless it be the result of observation and experimentation, and is thought to contain a newly discovered fact worthy of record.

---

